# Arthroscopic scapholunate ligament repair and dorsal capsulodesis with suture anchor in acute and subacute scapholunate dissociation

**DOI:** 10.1186/s13018-023-04148-y

**Published:** 2023-09-05

**Authors:** Yu-Cheng Lee, Yin-Chuan Shih, I-Ning Lo, Jui-Tien Shih

**Affiliations:** 1 Department of Orthopedic Surgery, National Taiwan University Hospital, Hsin-Chu Branch, Hsinchu, 300 Taiwan; 2 Department of Orthopedic Surgery, National Taiwan University Hospital, Taipei, 100 Taiwan; 3 Department of Orthopedic Surgery, En Chu Kong Hospital, New Taipei City, Taiwan; 4 Department of Orthopedic Surgery, School of Medicine, National Taiwan University, Taipei, Taiwan; 5 Department of Biomedical Engineering, Yuanpei University of Medical Technology, Hsinchu, Taiwan; 6 Department of Orthopaedics and Traumatology, Taipei Veterans General Hospital, Taipei, 112 Taiwan; 7 Department of Orthopaedics, School of Medicine, National Yang Ming Chiao Tung University, Taipei, 112 Taiwan; 8 Department of Orthopedic Surgery Centre for Sports Medicine Armed Forces Taoyuan General Hospital, 168, Zhongxing Rd., Longtan Taoyuan, 325 Taiwan

**Keywords:** Scapholunate instability, Wrist arthroscopy, Ligament injuries

## Abstract

**Purpose:**

The objective of this study was to investigate the potential of arthroscopic scapholunate ligament repair and dorsal capsulodesis with suture anchor as a treatment option for patients experiencing symptomatic acute and subacute (< 3 months) scapholunate instability.

**Methods:**

From Jan. 2017 to Jan 2020, 19 wrists with acute or subacute tears of the SL ligament with symptomatic instability were treated with arthroscopic SL repair and dorsal capsulodesis with a suture anchor. The average time from injury to operation was 8.8 weeks (range, 4–11 weeks) and the regular follow-up mean duration at our clinic was 26.5 months (range, 24–32 months). The pain score according to the visual analog scale, wrist range of motion, grip strength, radiographic outcomes and functional outcomes according to the Modified Mayo Wrist Score (MMWS) were evaluated preoperatively and postoperatively during the follow-up period.

**Results:**

All 19 patients had rupture and dissociation of the SL ligament in the radiocarpal joint. The total arc of wrist motion in the flexion–extension plane loss averaged 5.1° (*P* > .01).The Wilcoxon signed-rank test was used to compare the results: grip force improved significantly with 14.7% improvement of that on the normal side (*P* < .01); the postoperative MMWS was significantly better than the preoperative scores (*P* < .01). Of 19 patients of the series, 18 patients (94.7%) achieved good or excellent results according to the MMWS and 16 patients (84.2%) resumed their previous activities. Only one patient (5.3%) had residual laxity of the scapholunate ligament joint at 15 months of follow-up.

**Conclusions:**

At a minimum of two years of follow-up, patients with acute or subacute symptomatic dissociation of scapholunate ligament instability who underwent arthroscopic scapholunate ligament repair and dorsal capsulodesis with suture anchor treatment had satisfactory results.

**Level of Evidence:**

Level IV, case series.

## Introduction

Scapholunate (SL) ligament injury, which is the most common cause of carpal instability, can lead to pain, loss of wrist function and progressive wrist arthritis [1].

Early traditional open repair with capsulodesis have been shown to yield satisfactory results in some studies [2–7]. However, the wrist arthroscopic surgery is a less soft-tissue damaging method that not only to identify SL ligament tears but could be used to repair the SL ligament and wrist dorsal capsulodesis [8–10].

There are few studies to demonstrate the outcomes of arthroscopic-assisted primary repair and dorsal capsulodesis in patients with acute or subacute phase SL ligament injury [11–13]. Those studies produced inconsistent results among patients treated with the arthroscopic-assisted SL ligament primary repair. Therefore, we aim to evaluate the minimum 2-year follow-up clinical functional results in patients with repairable acute or subacute phase SL ligament tears treated with arthroscopic-assisted SL ligament suture anchor repair and dorsal capsulodesis. Our hypothesis is that arthroscopic-assisted SL ligament primary repair is a minimal invasive and reliable technique in patients with SL ligament acute or subacute phase tears (< 3 months).

## Methods

Approval for this study was obtained from our hospital’s institutional review board. During the period from January 2017 to January 2020, we treated 19 young patients sustained symptomatic SL ligament tear within 3 months of an injury. (There is a definition by literatures that acute phase is within 4 weeks, subacute is 4 weeks to 6 months and chronic is over 6 months from the initial injury [7, 14]). These patients underwent arthroscopic SL ligament repair and dorsal capsulodesis with a suture anchor (CONMED Y-note 1.3 mm). All 19 recruited patients’ SL ligament instability were assessed through the scaphoid shift test and SL stress test, and the results of these tests were compared to those of the uninjured wrist preoperatively and at minimum 2 years postoperatively. Furthermore, the clinical outcome, including visual analog scale (VAS) pain score, wrist range of motion (ROM), grip strength, and wrist functional outcome (Modified Mayo Wrist Scores) were measured preoperatively and postoperatively as well.

Plain radiographs were obtained from all patients, including loaded clenched fist films and unloaded ordinary plain films. Scapholunate widening was assessed by one surgeon and one radiologist. As clinical examination revealed ligament insufficiency, magnetic resonance imaging was routinely performed to identify the SL ligament rupture, bone edema and other associated acute injury. All patients underwent wrist arthroscopy not only to confirm the diagnosis but also to assess associated injury or the occurrence of the chondral lesion. Postoperative plain radiographs were evaluated for the stability between SL ligaments as well. Patients with chronic SL ligament injury (> 3 months), dorsal intercalated segmental instability, carpal bone fractures, or degenerative changes in their radiocarpal or midcarpal joints were excluded from this study.

### Surgical technique

**Fig. 1:**
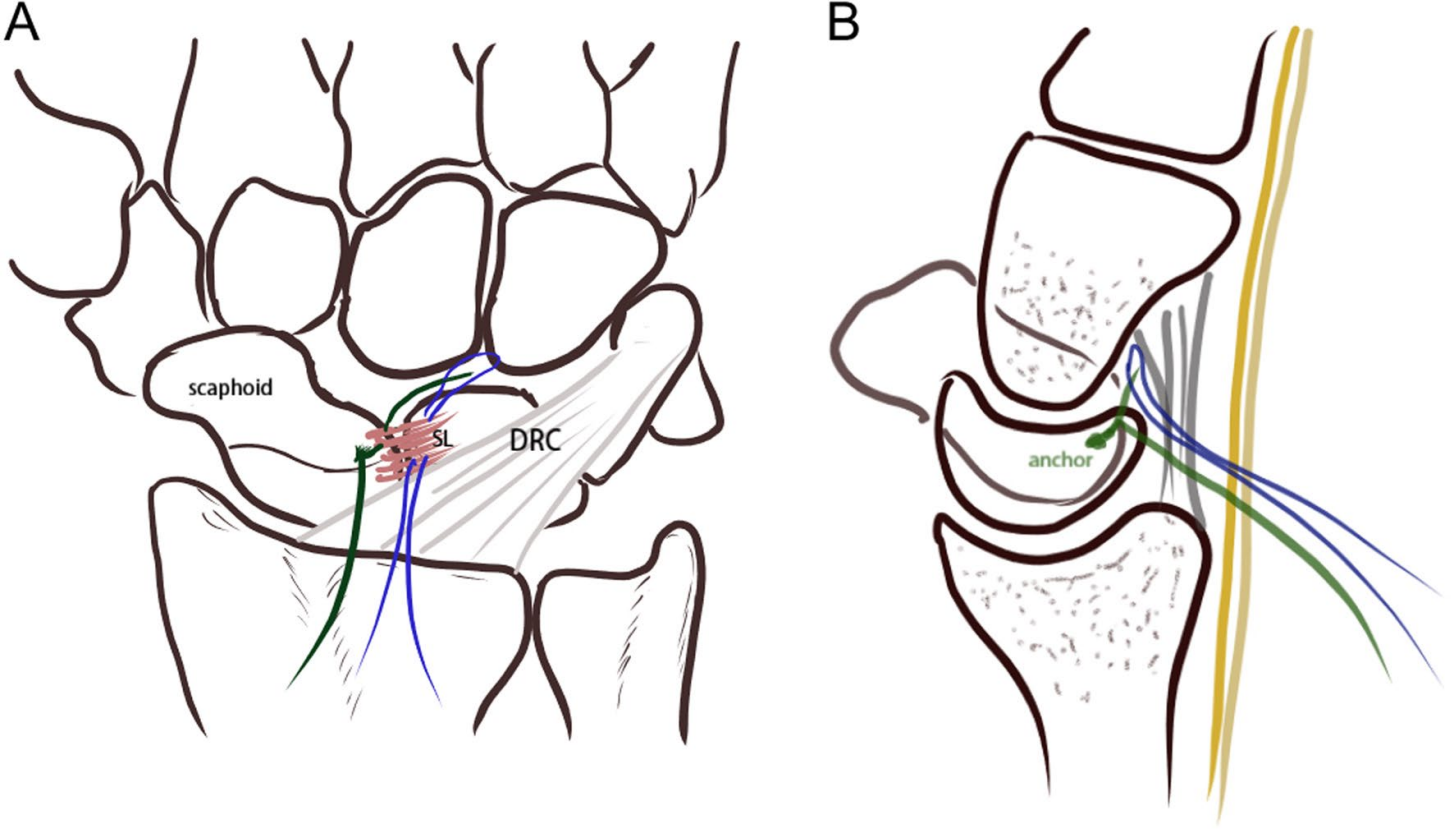
**A, B** The first limb of the suture anchor pass through residual SL ligament from the scaphoid or the lunate by needle to the midcarpal joint. The same limb passage with the 2–0 nylon loop via the 3, 4 portal through dorsal capsule, DRC ligament and residual SL ligament. Then, tie the two limbs over the dorsal capsule with the SL ligament and DRC ligament

**Fig. 2:**
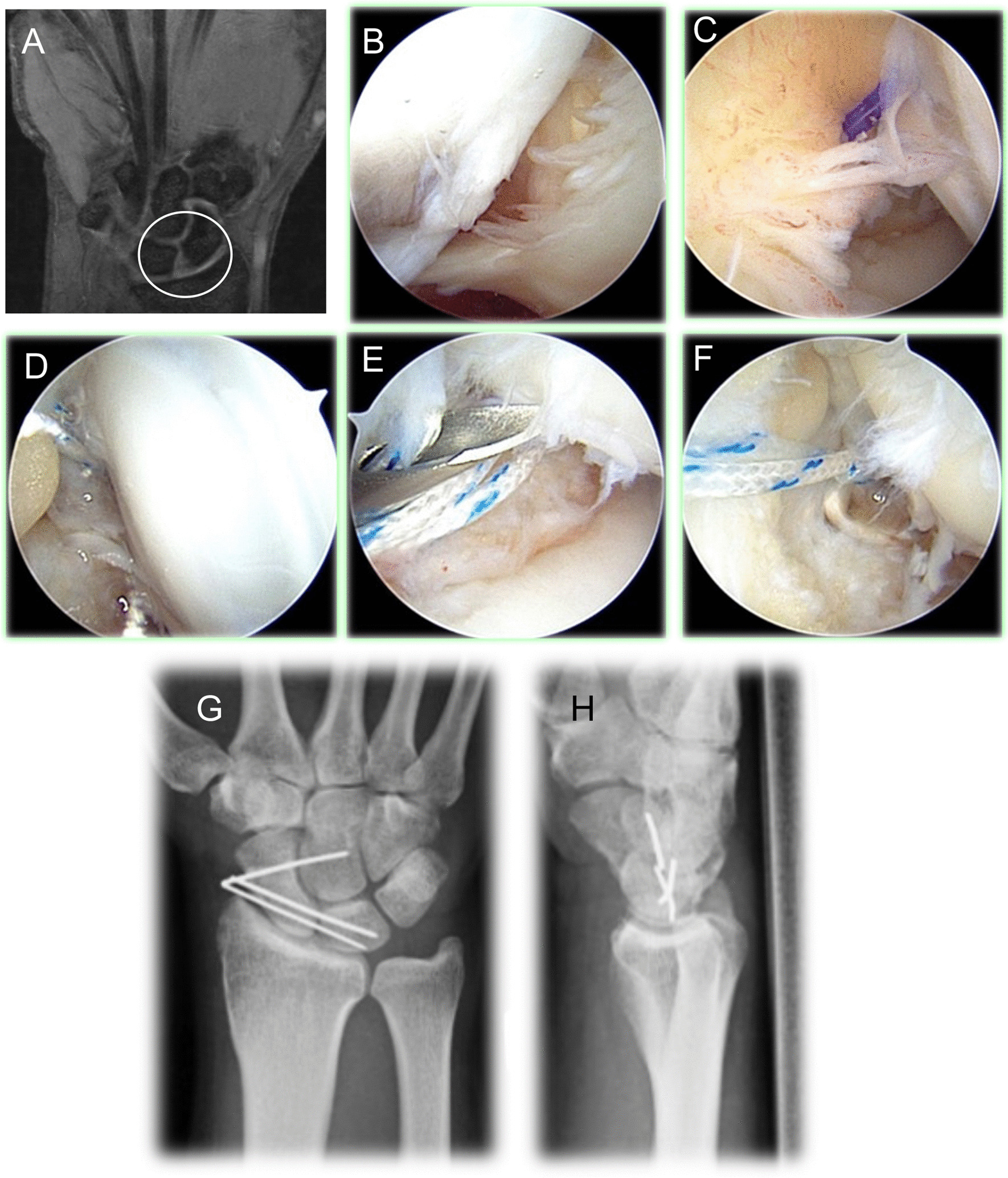
A 25-year-old male experienced acute tear of SL ligament from scaphoid bone (Anderson-Garcia-Elias type 1) (**A, B**). A loop suture via needle catches the dorsal capsule, partial dorsal radiocarpal ligament and SL ligament (ulnar site) to midcarpal joint from 3–4 portal (**C**). A 1.3 mm all suture anchor applied to proximal dorsal part of scaphoid (**D**). A limb of fiberwire was sent to midcarpal joint and just pass through the loop suture (**E**). The limb of fiberwire was pulled back to radiocarpal joint by loop suture, the SL ligament was suture back to scaphoid bone (**F**). The SL and SC joints were fixed by K-wires (**G, H**)

We used standard wrist arthroscopic equipment with finger trap, wrist tower distraction, and an upper arm tourniquet to ensure clear visualization during surgery. All patients received general anesthesia or axillary nerve block. A dorsal approach via 3–4, 6R, midcarpal radial (MCR), and midcarpal ulnar (MCU) portals were created. We explored and identified the SL ligament injury type according the Andersson-Garcia-Elias classification [15] from 6R portal first. The quality of the SL ligament was evaluated by probing through the 3–4 portal, and other intra-articular pathologic conditions were also examined. Findings such as step-off or dissociation between the scaphoid and lunate were assessed by pushing the scaphoid and probing the SL interval from the MCR portal, to identify the grading of instability according to the Geissler classification [16]. We applied mini-all suture anchor (CONMED Y-note 1.3 mm) over the dorsal proximal scaphoid or lunate via 3–4 portal after SL ligament tear was confirmed. One limb of suture anchor passed through the scaphoid part or lunate part of SL ligament by needle and then reached midcarpal joint. Subsequently, the same limb of the anchor threaded through the 2–0 nylon loop protected by needle which passing the SL ligament through the dorsal radiocarpal (DRC) ligament and then pulled back to radiocarpal joint. (Fig. 1) After reducing the SL joint and fixing with K-wires, we secured the knots over the dorsal capsule with SL ligament and DRC ligament by utilizing the 3/4 portal and conducting meticulous dissection of the extensor tendons to avoid any soft-tissue impingement. After repairing the SL ligament, the stability and alignment of the SL joint would be re-evaluated from the MCR portal. Under fluoroscopy, the SL joint was fixed by two 0.8 mm K-wires; meanwhile, the scapho-capitate joint would be fixed as well for more carpal row stability [17] (Fig. 2).

Arthroscopic treatment procedures were the same in all patients; after confirming acute or subacute SL ligament tears, the other structures of the radiocarpal joint were also evaluated.

After the operation, we applied a plaster cast for 8 weeks, and patients began a rehabilitation program that included gentle wrist motion and occupational therapy once the cast was removed. The K-wires were removed after 12 weeks postoperatively.

### Statistical analysis

The normality of the data was verified using the Kolmogorov–Smirnov test, which showed that the data were nonparametric. The Wilcoxon signed-rank test was used to compare preoperative and postoperative values. Statistical analyses were demonstrated by using SPSS for Windows version 24 (SPSS Inc, Armonk, NY). Statistical significance was set at *P *< 0.05.

## Results

This study comprised 16 men and 3 women, with an average age of 24.1 years (range: 17–27 years) (Table 1). These patients presented with wrist pain that hindered their ability to perform daily activities or exercise. All patients experienced a traumatic incident before developing symptoms in their wrists. The average period from the time of injury to surgery was 9 weeks (range: 4–11 weeks).

**Table 1:** Demographics and Clinical Characteristics of Patients

Characteristic	Patient number = 19
Male/Female	16/3
Dominant hand	10
Mean age	24.1 (17–27)
Duration symptoms	9 (4–11) weeks
Follow-up	26.5 (24–32) months
*Andersson-Garcia-Elias classification*	
Type 1 (lateral avulsion off scaphoid)	17
Type 2 (medial avulsion off lunate)	2
Associated injury	
TFCC tear	5
RSC ligament injury	3

*TFCC* Triangular fibrocartilage complex, *RSC* Radioscaphocapitate

In our series, all 19 patients presented with tenderness in the region of the dorsal wrist, as well as the positive results of both the scaphoid shift test (Watson test) and SL ballottement test. The clenched fist radiographs showed a wide interval (> 3 mm) in the SL joint of 15 patients. To confirm the diagnosis of acute or subacute tears of the SL ligament, evaluate ligament quality, and detect other lesions in radiocarpal or midcarpal joints, all patients underwent wrist arthroscopy surgery. Via the midcarpal portals, a step-off with Geissler grade III SL ligament tear was found in all wrists. The scaphoid avulsion SL ligament tear (type 1) was found in 17 patients while lunate avulsion SL ligament tear (type 2) was found in 2 patients, according to the Andersson-Garcia-Elias classification [15].

All 19 patients in our study underwent arthroscopic repair of the SL ligament and dorsal capsulodesis with a suture anchor. These patients were regularly followed up at our clinic for an average of 26.5 months (range, 24–32 months). At the minimum 2 years follow-up, patients’ VAS and grip force improved significantly (*P *< 0.01), with a 14.7% strength increase from pre-operative phase to postoperative phase. The mean MMWS was 90.1 points (range, 70–100), and the Wilcoxon signed-rank test revealed that postoperative scores were significantly better than preoperative scores (*P *< 0.01). At the 2-year follow-up, there was an average loss of 5.1° in the total arc of wrist motion in the flexion–extension plane (Table 2). In our series, 5 patients (26%) were found TFCC peripheral tear, while 3 patients (16%) presented with radioscaphocapitate (RSC) ligament injury. During the surgery, these patients underwent the TFCC repair and RSC ligament thermal shrinkage, respectively.

**Table 2:** Clinical Outcomes

	Pre-operation	Post-operation	
VAS pain score	5.1	1.6	*p* < 0.01
Grip strength % of healthy side	58.6%	73.3%	*p* < 0.01
Wrist flexion–extension arc	114.2	109.1	> 0.1
Modified Mayo Wrist Score	60.3	90.1	*p* < 0.01
Excellent	0	12	
Good	0	6	
Fair	7	1	
Poor	12	0	

Postoperative radiographs revealed no change in the SL angle, and clenched fist wrist radiographs showed the absence of a gap over the SL joint in 18 patients. Other than recurrent SL instability in one patient, there were no other complications among the 19 patients. One patient (5.3%) exhibited laxity of the SL joint with a positive Watson test and SL joint diastasis in clenched fist view after 15 months following the surgery. Therefore, the patient underwent an SL ligament reconstruction surgery with palmaris longus tendon graft via open-dorsal approach. During the operation, limited vascularity remnants with fibrous scar tissue was found in patient’s SL joint. There was symptoms improvement and satisfactory results in patient’s VAS and MMWS after a year follow-up.

## Discussion

As early diagnosis of acute SL ligament tear is challenging, wrist arthroscopy is recommended as a valuable tool not only for confirming the diagnosis and developing appropriate treatment modalities for SL ligament injuries, but providing quality of ligaments and other associated ligament injury or osteochondral lesion [10, 18, 19]. The diagnosis and treatment in acute phase of SL ligament rupture is critical due to the remnant of ligaments deteriorated with time and bony malalignment or degeneration in chronic SL instability [7, 12]. Thus, some literature demonstrated the ideal time for repairing the SL ligament is within 4–6 weeks after the injury. However, the time to repair could be extended in Anderson-Garcia-Elias type 1 and 2 which ligaments avulsed from scaphoid and lunate, with better vascularization and ligament integrity [7, 20, 21].

Numerus procedures have been demonstrated to repair acute phase SL dissociation, including open approach to reinsertion the SL ligament by transosseous suture or anchor repair with dorsal capsulodesis and K-wire fixation [6]. Several studies have published that the open repair in acute phase SL ligament rupture was a reliable method with satisfactory results, but there are associated morbidity such as stiffness [22–25]. Rosati et al. reported 72.2% excellent results in 18 patients undergoing open SL ligament repair treatment in acute phase SL dissociation under 32-month mean follow-up. Minami et al. also published a 17-patient series that early open repair treatment in SL ligament tear has better radiographic outcome than later phase surgery. However, the postoperative wrist showed less flexion motion (55 degrees) compared to uninjured wrist (70%). In addition, the secondary stabilizer damaging and posterior interosseous nerve injury during dorsal wrist approach would impair patient’s carpal stability and proprioception of SL ligament as well [8, 9]. Nevertheless, there have been few studies reported that arthroscopic SL ligament repair could reproduce the similar treatment effect as open approach. Literature by Anderson et al. stated that arthroscopically-assisted SL capsuloplasty and suture may not be feasible for all patients, especially when the ligament has avulsed off the bone which may result in a situation where there is no ligament remnant remaining on one side, making the mentioned surgical procedure impractical [7]. Weiss et al. demonstrated that arthroscopic debridement with K-wire fixation has proven to be effective in providing reliable pain relief for patients with partial tears while patients with complete ligament tears experienced a worse outcome [13, 26].

Recently, Carratalá et al. [27] published the encouraging results of 19 patients with acute phase SL ligament injury treated with arthroscopic-assisted repair and dorsal capsulosesis. After 12 months of follow-up, they obtained 79% excellent or good results with significant improvement in VAS score, grip strength and wrist motion. The wrist ROM showed a reduction of 10 degrees when compared with that in the contralateral wrist.

## Limitation

There are some limitations in this study, including a small sample size and potential interobserver variability in physical findings. Besides, there were no control group patients with conservative treatment or traditional open procedure.

## Conclusion

The analytical results suggest that repairing the SL ligament with a suture anchor in patients with acute or subacute phase SL ligament dissociation can be a reasonable and minimal invasive surgical treatment, with satisfactory outcomes observed at a minimum follow-up of 2 years.

## Data Availability

The dataset supporting the conclusions of this article is included within the article.

## Abbreviations

SL: Scapholunate
MMWS: Modified mayo wrist score
VAS: Visual analog scale
MCR: Midcarpal radial
MCU: Midcarpal ulnar
K-wires: Kirschner-wires
ROM: Range of motion
TFCC: Triangular fibrocartilage complex
RSC: Radioscaphocapitate
DRC: Dorsal radiocarpal
